# One-Step Synthesis and Performance Evaluation of a Sucrose–Glyoxylic Acid Wood Adhesive

**DOI:** 10.3390/ma18235386

**Published:** 2025-11-28

**Authors:** Jiankun Liang, Yuqi Yang, Longxu Wu, Ningyuan Zuo, Qiuli Li, Tong Meng, Chuchu Chen, Huali Li, Caihong Long, Zhixian Song, Yulan Jian, De Li, Zhigang Wu

**Affiliations:** 1 College of Civil Engineering, Kaili University, Kaili 556011, China; dushimensheng@126.com; 2 College of Forestry, Guizhou University, Guiyang 550025, Chinawzhigang9@163.com (Z.W.); 3 School of Chemistry and Material Engineering, Zhejiang A&F University, Hangzhou 311300, China

**Keywords:** sucrose–glyoxylic acid wood adhesive, bonding performance, curing performance, thermal stability

## Abstract

Environmental and health concerns drive research into sustainable bio-based wood adhesives. This study utilized widely available and economical sucrose and glyoxylic acid as raw materials to prepare a wood adhesive via a one-step method. The effects of glyoxylic acid content on the adhesive structure, properties, and plywood application performance were statistically investigated. The results demonstrated successful esterification and acetalizations between glyoxylic acid and sucrose, forming a dense three-dimensional cross-linked network that enhanced bonding performance, water resistance, and thermal stability. At 40% glyoxylic acid content, the adhesive exhibited optimal comprehensive properties: the wet shear strengths of 1.39 MPa (63 °C) and 1.17 MPa (93 °C) that substantially exceeded GB/T 17657-2022 requirements. This study provides novel insights and a practical foundation for high-value sucrose utilization and green wood adhesive development.

## 1. Introduction

Wood adhesives critically determine the strength and durability of wood-based panels. Formaldehyde-based resins (PF, UF, MUF) have long dominated due to excellent bonding and mature processes [1,2,3,4], but their reliance on fossil resources and release of formaldehyde/VOCs pose serious health risks [5,6]. With growing environmental concerns and resource constraints, developing sustainable, high-performance bio-based alternatives has become an urgent priority. In this context, various biomass resources, such as proteins [7,8,9,10,11], lignin [12,13,14,15], tannins [16,17,18], starch [19], and sugars [20,21], have been widely explored for the preparation of environmentally friendly adhesives. Among them, sucrose has shown great potential due to its wide availability, renewability, non-toxicity, and low cost. Sucrose is a disaccharide composed of glucose and fructose linked by a glycosidic bond and is one of the most abundant natural carbohydrates in the world, with stable supply and minimal price fluctuations, and it is non-toxic and harmless [22]. From a molecular structure perspective, sucrose molecules are rich in multiple hydroxyl groups, providing abundant reactive sites for chemical modification. Theoretically, these hydroxyl groups can undergo cross-linked reactions with other functional groups to form high molecular weight polymers with a three-dimensional network structure, thus meeting the basic conditions for adhesives.

Recent years have witnessed extensive research on sucrose-based adhesives. One approach involves catalytic dehydration of sucrose with ammonium dihydrogen phosphate to produce 5-hydroxymethylfurfural for plywood that meets wet shear strength standards [22]. Alternative strategies include cross-linking sucrose with tannin to achieve excellent bonding performance [23]. Molecular modification—particularly oxidation—has emerged as a key strategy for overcoming performance limitations, enabling precise control over functional group reactivity. For instance, ammonium persulfate oxidation converts sucrose hydroxyl groups into aldehydes, which subsequently undergo Schiff base reactions with hexamethylenediamine to form three-dimensional networks, achieving a wet shear strength of 0.98 MPa [24]. Similarly, selective oxidation of sucrose’s vicinal diols with sodium periodate produces polyaldehyde derivatives that, when combined with aminated cellulose, attain 1.48 MPa wet shear strength on epoxy-functionalized wood surfaces [25]. These findings confirm that introducing aldehyde groups substantially enhances interactions between sucrose, cross-linking agents, and wood substrates. Nevertheless, current oxidation systems face challenges in completely removing residual oxidants, raising environmental concerns, while issues of cost, controllability, and reaction complexity persist. Developing simpler and more efficient curing systems remains an important research direction.

Glyoxylic acid, as a binary active molecule with both aldehyde and carboxyl groups, provides a new idea for the efficient modification of sucrose. Compared with traditional oxidants, the modification mechanism of glyoxylic acid has unique advantages: on one hand, its aldehyde group can react with the hydroxyl groups in sucrose molecules to form hemiacetal or acetal structures, which then dehydrate to form ether bonds under mild conditions, achieving preliminary cross-linked of the molecular chains [26]; on the other hand, the carboxyl group can undergo esterification with the hydroxyl groups of sucrose, introducing hydrophobic ester bonds to provide certain water resistance [27]. In addition, glyoxylic acid itself is derived from biomass conversion processes and has the characteristics of being non-toxic and biodegradable, in line with the concept of green chemistry.

Based on this foundation, the present study proposes, for the first time, to use sucrose and glyoxylic acid—both abundant and cost-effective—as the main raw materials to prepare a novel sucrose–glyoxylic acid wood adhesive with high water resistance that meets industrial application requirements via a one-step method. This pioneering work not only provides a new approach for the high-value utilization of sucrose but also offers theoretical support for the performance optimization of bio-based adhesives. As the first investigation into the sucrose–glyoxylic acid adhesive system, it holds great theoretical and practical significance for promoting the green transformation of the wood industry.

## 2. Materials and Methods

### 2.1. Materials

Sucrose was sourced from Yuanye Biotechnology Co., Ltd. (Shanghai, China). Glyoxylic acid (50% concentration) was procured from Anergy Chemical Reagent Co., Ltd. (Nanjing, China). Poplar veneer (*Populus* spp., with a moisture content of 8–10% and a density of approximately 0.4 g/cm^3^), measuring 400 mm × 400 mm and 1.5 mm in thickness, was obtained from Shuyang, Jiangsu Province, China.

### 2.2. Preparation of Sucrose-Glyoxylic Acid Adhesive

At room temperature, sucrose was dissolved in water to prepare a 50% aqueous solution. After complete dissolution, glyoxylic acid was added to the solution at 20%, 40%, 60%, and 80% of the mass of sucrose (the dry mass ratio), and the mixtures were stirred until uniform. The samples were designated as S20, S40, S60, and S80, respectively. Additionally, the adhesive sample without added glyoxylic acid was labeled as S0. The specific formulations are shown in Table 1. The viscosities of the obtained adhesives were 16.1, 15.6, 13.5, 12.9, and 12.1 mPa·s, respectively.

### 2.3. Plywood Preparation and the Testing of Bonding Strength

In the laboratory, a three-layer poplar plywood was manufactured by applying the adhesive to one side of the poplar veneer at a loading rate of 140 g/m^2^. The plywood assembly was completed within 10 min. Following assembly, the plywood underwent an initial 10 min cold-pressing, succeeded by hot-pressing. The hot-pressing conditions were as follows: the hot-pressing temperature was maintained at 200 °C, the unit hot-pressing pressure was 1.0 MPa, and the hot-pressing duration was 5 min. The plywood panel were prepared and conditioned in the laboratory at 20 ± 2 °C and relative humidity of 65 ± 5% for 1 day. The bonding strength of the plywood was assessed in compliance with the national standard GB/T 17657-2022 [28]. The final strength is the average of the 12 samples.

### 2.4. Residue Rate Test

Following the curing process, the cured adhesive was manually ground using a mor-tar and pestle to obtain a fine powder, with 5 replicates prepared per sample. This powder was subsequently enclosed in filter paper and submerged in water maintained at a temperature of 63 ± 3 °C for a duration of 3 h. Post immersion, the residual solid was isolated through filtration and then dried until its weight remained unchanged. The residue rate of the cured adhesive was determined by comparing its mass prior to and following the immersion process.

### 2.5. Moisture Absorption Test

Approximately 2 g of cured adhesive powder was placed in an aluminum foil container and conditioned in a constant temperature and humidity chamber at 30 °C and 85% relative humidity. The sample was weighed every 2 h until mass equilibrium was reached (mass change < 0.1% within 2 h). 5 replicate tests were performed per sample.

### 2.6. Fourier Transform Infrared Spectroscopy (FTIR) Test

The analysis was conducted using a Varian 1000 IR spectrometer (Varian, Palo Alto, CA, USA) in transmittance mode. The wavenumber range was set from 400 to 4000 cm^−1^, with a resolution of 4 cm^−1^ and 32 scans. The test environment was maintained at an indoor temperature of 25 °C and a relative humidity of 60%.

### 2.7. Differential Scanning Calorimetry (DSC) Test

The differential scanning calorimeter employed for the test was a DSC 204 F1 manufactured by Netzsch, Rodgau, Germany. The test was conducted with the following parameters: a temperature range of 25–250 °C, a heating rate of 10 °C/min, under nitrogen protection, and with a sample mass between 5 and 8 mg.

### 2.8. Thermogravimetric (TG) Test

The cured adhesive powder was analyzed via thermogravimetric analysis (TGA) using a TG 209 F3 instrument from Netzsch, Rodgau, Germany. The test was performed under nitrogen atmosphere with a heating rate set at 10 °C/min and temperatures from 30 to 700 °C.

### 2.9. X-Ray Diffraction (XRD) Test

The measurements were conducted utilizing an Ultima IV X-ray diffractometer (Tokyo, Japan), equipped with a Cu target (λ = 0.154060 nm). The 2θ scanning range was from 5 to 60°, with a step increment of 0.02° and a scanning velocity of 5°/min. The tube settings were configured at a current of 120 mA and a voltage of 40 kV.

### 2.10. Scanning Electron Microscopy (SEM) Test

Following the application of a gold sputter-coating to the cross-sectional surface of the cured adhesive layer, the morphology was examined using a Zeiss GeminiSEM 300 scanning electron microscope from Tokyo, Japan.

### 2.11. Statistical Analysis

The data were processed using Excel 2021 and Origin 2024 software, and the significance of differences was judged via the one-way analysis of variance (ANOVA) (*p* < O.05), the error bars represent the standard deviation.

## 3. Results and Discussion

### 3.1. FTIR Analysis

**Figure 1:**
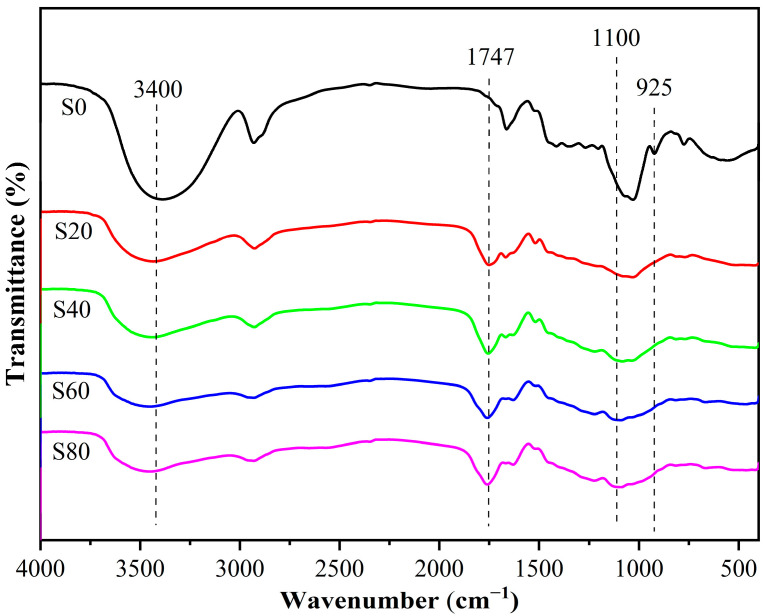
FT-IR curves of sucrose-glyoxylic acid adhesive.

As depicted in Figure 1, the broad absorption peak near 3400 cm^−1^ is attributed to the hydroxyl (-OH) groups in sucrose molecules. Upon the addition of glyoxylic acid to the system, this peak significantly diminishes. This reduction indicates that a substantial number of hydroxyl groups in sucrose molecules have engaged in chemical reactions with glyoxylic acid during the curing process, leading to a decrease in the hydroxyl group count and, consequently, a decline in the intensity of the absorption peak. Moreover, the characteristic absorption peak of the hydroxymethyl (-CH_2_OH) group in sucrose molecules, which is located at 925 cm^−1^ [23], also vanishes after the introduction of glyoxylic acid. This further corroborates that the hydroxyl groups in sucrose are consumed during the reaction with glyoxylic acid, resulting in a structural alteration of the hydroxymethyl group.

**Scheme 1:**
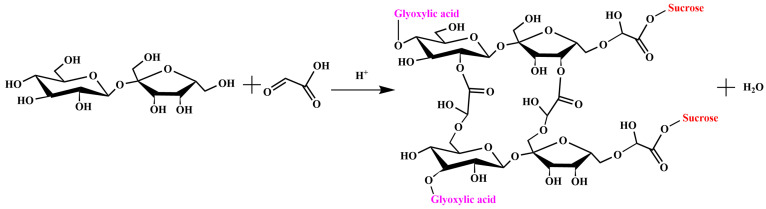
The possible reaction mechanism of glyoxylic acid and sucrose.

Of particular significance is the emergence of two new absorption peaks following the addition of glyoxylic acid. The peak at 1747 cm^−1^ corresponds to the C=O stretching vibration of the ester group (-COO-), indicating that esterification has occurred between sucrose and glyoxylic acid, leading to the formation of ester bonds. Additionally, the peak at 1100 cm^−1^ is associated with the C-O-C stretching vibration in the acetal or hemiacetal structure formed from the addition reaction between the aldehyde group (-CHO) and the hydroxyl group [29,30]. The presence of these new peaks demonstrates that the cross-linked structure resulting from the curing of sucrose and glyoxylic acid primarily consists of ester bonds and acetal structures (Scheme 1).

### 3.2. XRD Analysis

**Figure 2:**
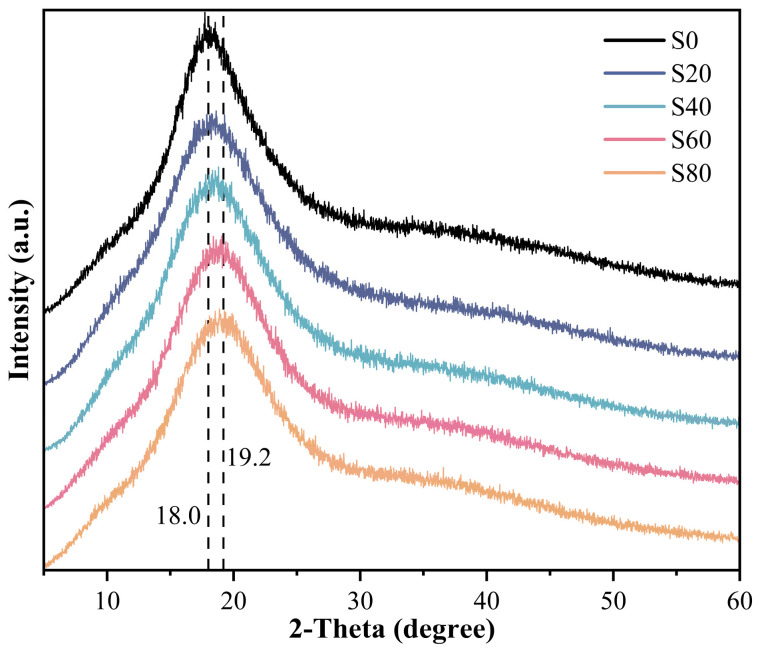
XRD curves of sucrose-glyoxylic acid adhesive.

As shown in the XRD patterns in Figure 2, the unmodified sample S0 exhibits a sharp crystalline diffraction peak at around 18.0°, which is a typical characteristic of the crystalline structure of sucrose. This indicates that the S0 has a high degree of crystallinity, with a well-ordered molecular arrangement and a good crystalline structure. However, with the addition of glyoxylic acid (from S20 to S80), the original sharp crystalline peak has significantly weakened, replaced by a broad and flat “hump-like” diffuse peak. This typical “hump peak” is a hallmark of amorphous materials, indicating that the crystallinity of the samples gradually decreases and the molecular arrangement becomes more disordered. It is also worth noting that the diffraction peak shifts from 18.0° to 19.2°, a shift that further illustrates the structural changes in the samples. These transformations demonstrate that effective chemical reactions have occurred between glyoxylic acid and sucrose molecules. During the reaction, the addition of glyoxylic acid disrupts the regular hydrogen bond arrangement and crystalline structure of sucrose molecules, breaking the orderly arrangement of sucrose molecules and forming an amorphous, three-dimensional cross-linked polymer network. Glyoxylic acid not only alters the structure of sucrose but also fundamentally changes the structure of the adhesive. This structural change will further affect the macroscopic properties of the adhesive, such as its mechanical strength, flexibility, and water resistance.

### 3.3. SEM Analysis

**Figure 3:**
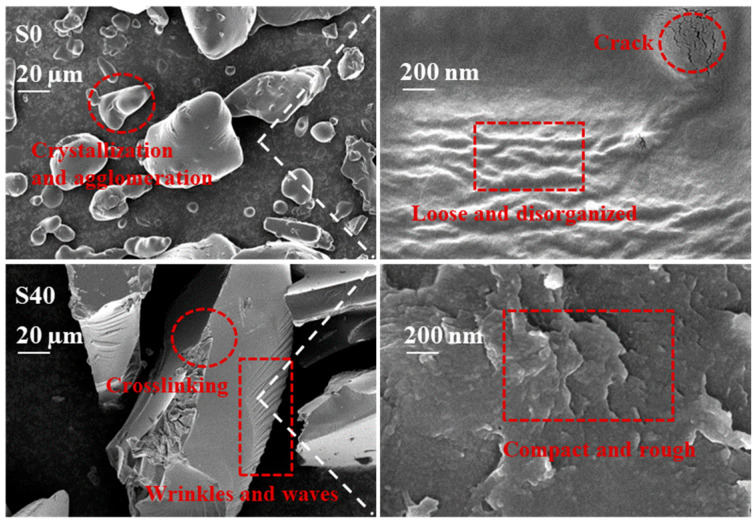
SEM curves of cured sucrose-glyoxylic acid adhesive.

The SEM results shown in Figure 3 clearly reveal the changes in the microstructure of sucrose-based adhesives before and after modification with glyoxylic acid. The unmodified sucrose particles exhibit a rounded shape, with an uneven and irregular size distribution. Their cross-sections are loose and cracked. This structure indicates that the unmodified sucrose molecules are primarily held together by hydrogen bonds, existing in a relatively loose aggregated state without regularity.

In contrast, after modification with glyoxylic acid, the adhesive particles display a regular, block-like cross-linked structure. The cross-sectional surface features distinct wrinkles and wavy patterns, and the texture is more compact. This indicates that chemical reactions have occurred between glyoxylic acid and sucrose, leading to significant changes in the molecular structure, such as cross-linked and adjustment of crystallinity, resulting in a more regular and dense morphology. This structural change allows the adhesive to absorb more energy during the fracture process, exhibiting higher fracture yield strength.

Combined FTIR and XRD analyses revealed that glyoxylic acid modification formed ester bonds and acetal structures while decreasing crystallinity, creating an amorphous cross-linked network. These microstructural changes enhanced mechanical properties—increasing energy absorption and fracture yield strength—and suggested improved adhesion and water resistance for practical applications.

### 3.4. DSC Analysis

**Figure 4:**
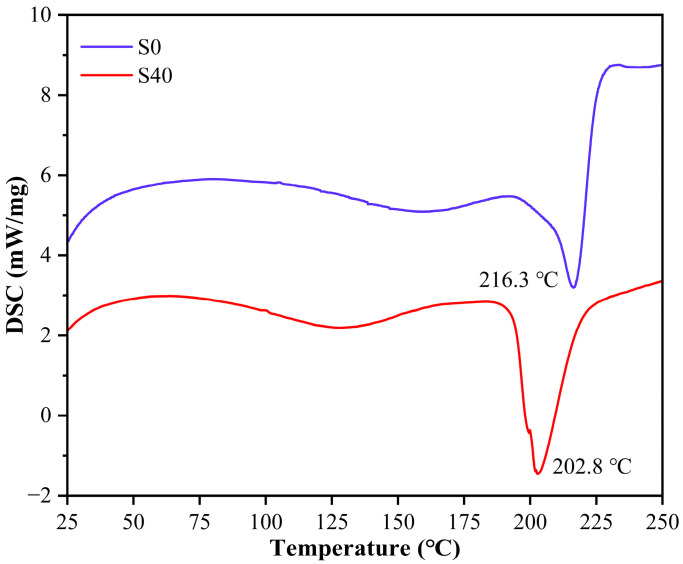
DSC curves of sucrose-glyoxylic acid adhesive.

The endothermic peaks in the DSC curves are significant indicators of the thermal transition behavior of materials, covering processes such as curing and thermal reactions. The DSC curves shown in Figure 4 reveal significant differences in the thermal transitions of sucrose-based adhesives before and after modification with glyoxylic acid. The curing peak of the unmodified S0 sample is at 216.3 °C, while the curing peak of the S40 sample, modified with 40% glyoxylic acid, is reduced to 202.8 °C. This result clearly indicates that modification with glyoxylic acid significantly lowers the curing temperature of sucrose-based adhesives. This reduction in curing temperature is likely due to the chemical reactions between glyoxylic acid and sucrose, which not only alter the molecular structure but also adjust the reaction activity, enabling the adhesive to initiate thermal behavior at a lower temperature. This is of great significance for reducing the curing temperature during adhesive use.

Additionally, both DSC curves show a gentle endothermic peak before 125 °C to 160 °C, and the endothermic temperature of the S0 sample is higher than that of the S40 sample. This phenomenon is likely closely related to the weak interactions such as hydrogen bonds and van der Waals forces between sucrose molecules and between sucrose and glyoxylic acid molecules. Within this temperature range, the breaking of these weak interactions absorbs heat, thus presenting a gentle endothermic characteristic. The addition of glyoxylic acid changes the material’s hygroscopicity and the way it binds with water, thereby affecting the starting temperature of the water removal process and causing the gentle peak to shift to the left. This change not only reflects the adjustment of the material’s microstructure but also implies potential alterations in its macroscopic properties.

In summary, modification with glyoxylic acid significantly enhances the thermal reactivity of sucrose-based adhesives, enabling them to form a stable bonding layer under lower heating conditions. This characteristic not only helps improve the bonding performance of the adhesive but also enhances its thermal stability. In practical applications, the modified adhesive can achieve efficient bonding effects at a lower curing temperature while maintaining good thermal stability. This is of great practical significance for improving production efficiency, reducing energy consumption, and enhancing product quality.

### 3.5. Thermal Stability Analysis

**Figure 5:**
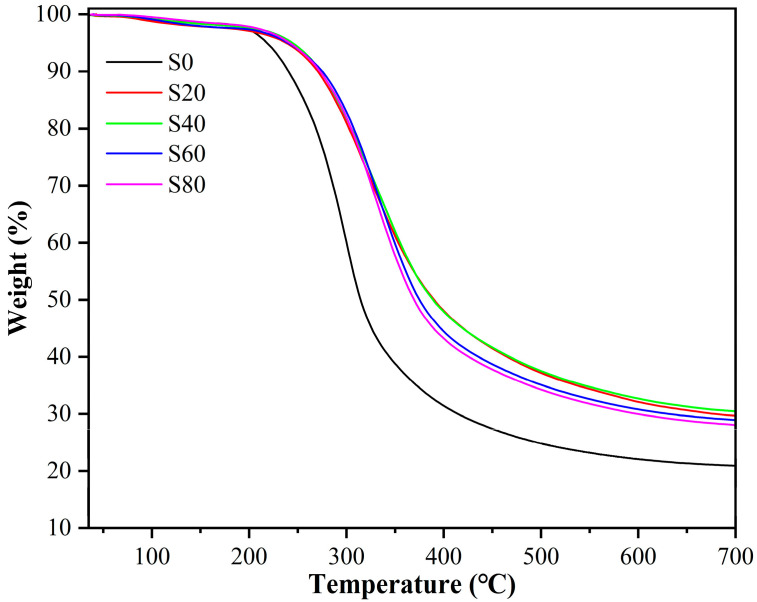
TG curves of sucrose-glyoxylic acid adhesive.

**Figure 6:**
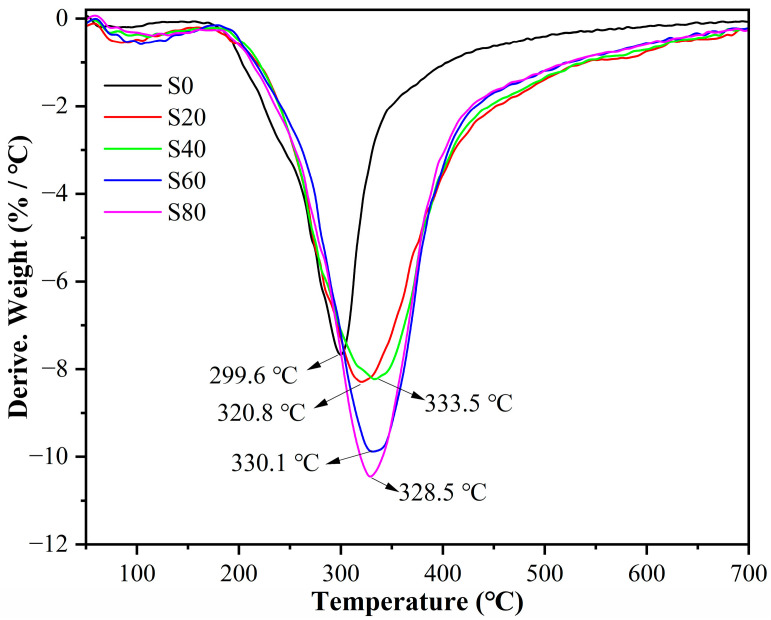
DTG curves of sucrose-glyoxylic acid adhesive.

TGA is a crucial method for evaluating the thermal stability and decomposition behavior of materials. By measuring the mass changes in materials during heating, it can reveal the thermal decomposition process and thermal stability of the materials. Figure 5 and Figure 6 show the TG/DTG curves of unmodified sucrose and samples modified with different amounts of glyoxylic acid. These curves clearly reveal the influence of glyoxylic acid on the thermal decomposition process of sucrose-based adhesives. The maximum decomposition peak of unmodified sucrose occurs at 299.6 °C. As a carbohydrate, sucrose undergoes a series of complex reactions in this temperature range, including dehydration, bond breaking, and the generation of small molecule volatiles [31]. These reactions lead to the breaking and decomposition of the sucrose molecular chain, ultimately forming a small amount of residual carbon. The residual carbon rate of the S0 sample is 20.9%, indicating that most of the unmodified sucrose decomposes into volatile products at high temperatures, with a small amount of carbon remaining.

With the introduction of glyoxylic acid, the decomposition behavior of the adhesive samples changes significantly. After adding 20% glyoxylic acid, the temperature of the maximum decomposition peak increases significantly to 320.8 °C. This indicates that the addition of glyoxylic acid significantly enhances the thermal stability of sucrose-based adhesives, causing significant thermal decomposition to occur at higher temperatures. Further increasing the amount of glyoxylic acid to 40% raises the maximum decomposition peak temperature to 333.5 °C. This shows that as the amount of glyoxylic acid increases, the thermal stability of sucrose-based adhesives is further enhanced, with higher thermal decomposition temperatures. When the amount of glyoxylic acid is increased to 60%, the maximum decomposition peak temperature slightly decreases to 330.1 °C. This indicates that further increasing the amount of glyoxylic acid does not continue to improve thermal stability but instead causes a slight decline. When the amount of glyoxylic acid is increased to 80%, the maximum decomposition peak temperature continues to drop to 328.5 °C. The maximum decomposition temperature increases significantly from 299.6 °C (S0) to 333.5 °C (modified with 40% glyoxylic acid), indicating that the addition of glyoxylic acid improves the thermal stability of the adhesive. The fundamental reason is that the carboxyl and aldehyde groups in glyoxylic acid react with the hydroxyl groups on sucrose molecules to form more stable acetal structures or ester linkages, constructing a three-dimensional network structure. This cross-linked structure enhances the interactions between molecular chains, increases the activation energy required for decomposition, and thus delays the onset of major structural breakdown to higher temperatures, significantly improving thermal stability.

The residual carbon content of the S0 sample is 20.9%. With the addition of glyoxylic acid, the residual carbon content of the adhesive increases significantly overall. The sucrose-based adhesive modified with 20% glyoxylic acid has a residual carbon content of 29.7%; the one modified with 40% glyoxylic acid has a rate of 30.5%; the one modified with 60% glyoxylic acid has a rate of 28.9%; and the one modified with 80% glyoxylic acid has a rate of 28.1%. These results further confirm the existence of the cross-linked structure. The dense cross-linked network tends to form a stable char layer during pyrolysis rather than completely decomposing into volatile small molecules. This stable char layer can effectively protect the internal structure of the adhesive, reducing mass loss at high temperatures and thus increasing the residual carbon content.

It is worth noting that when the amount of glyoxylic acid exceeds 40%, both thermal stability and residual carbon rate decrease. This indicates that there is an optimal range for the addition of glyoxylic acid. Excessive glyoxylic acid may lead to an overly dense cross-linked network, affecting the movement and reactivity of molecular chains, thereby reducing overall performance. Moreover, excess glyoxylic acid may introduce additional impurities or unstable factors, further affecting thermal stability and residual carbon rate. This finding is of great significance for optimizing the formulation of sucrose-based adhesives and enhancing their performance in high-temperature applications.

### 3.6. Bonding Performance Analysis

**Figure 7:**
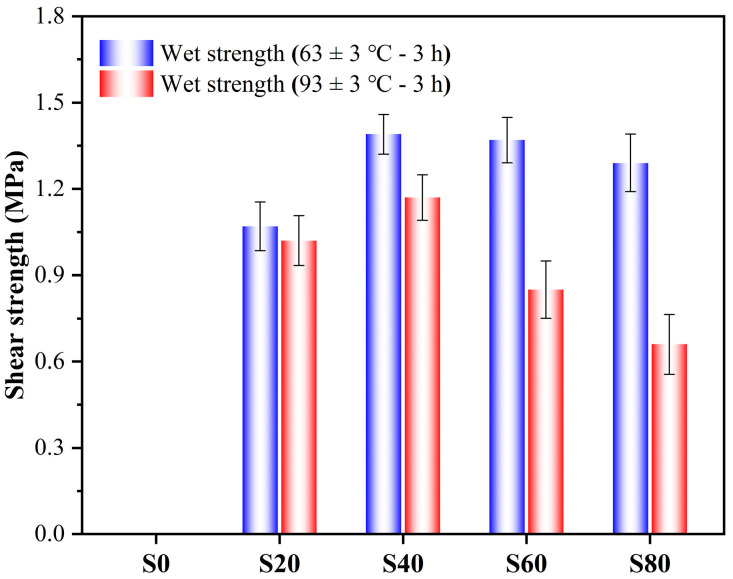
The shear strength of plywood prepared with sucrose-glyoxylic acid adhesive.

**Figure 8:**
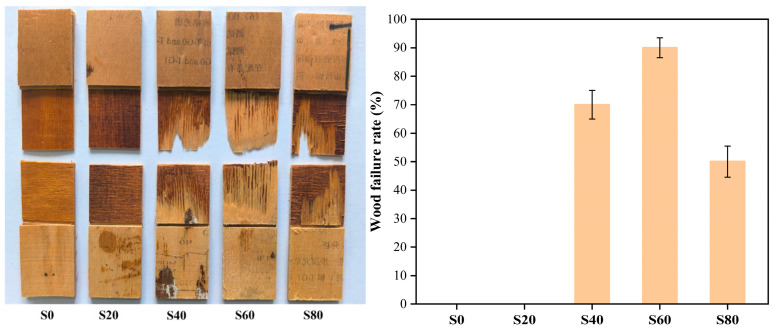
Wood breakage situation after shear test of plywood.

Figure 7 and Figure 8 show the bonding performance and wood failure characteristics of sucrose-based adhesives modified with different amounts of glyoxylic acid compared to unmodified sucrose (S0). The shear strength and wood failure rate of S0 are both 0. This indicates that under the curing conditions of 200 °C for 5 min, pure sucrose failed to form a sufficiently strong cross-linked structure to provide effective bonding strength. This result suggests that unmodified sucrose lacks the necessary mechanical properties for practical applications and cannot meet the requirements of an adhesive.

As the proportion of glyoxylic acid added increases gradually, the shear strength shows an overall trend of first increasing and then decreasing. This indicates that an appropriate amount of glyoxylic acid can effectively enhance the shear strength of the adhesive, but an excessive amount may lead to a decline in performance. Specifically, for the 20% glyoxylic acid formulation, the shear strength increases significantly. For the 40% glyoxylic acid formulation, the shear strength reaches its optimal value, with the warm water shear strength (63 °C—3 h) reaching 1.39 MPa and the boiling water shear strength (93 °C—3 h) reaching 1.17 MPa, both exceeding the requirements of the Chinese national standard GB/T 17657-2022. Combined with FTIR analysis, these performance improvements are attributed to the successful cross-linked reactions between glyoxylic acid and sucrose. The aldehyde group of glyoxylic acid likely forms an acetal structure with the hydroxyl group of sucrose, and its carboxyl group also participates in esterification reactions, together constructing a dense three-dimensional network structure. This network greatly enhances the cohesive strength of the adhesive itself and its bonding strength with the wood interface. This result further proves that glyoxylic acid reacts effectively with sucrose to form a denser and more stable cross-linked network. However, further increasing the amount of glyoxylic acid added leads to a decrease rather than an increase in shear strength. This may be because an excess of glyoxylic acid can alter the pH of the reaction system, causing sucrose to degrade under high-temperature conditions, generating sugar derivatives [32]. These derivatives increase impurities in the adhesive and inhibit the smooth progress of the main cross-linked reactions, thereby reducing the overall performance of the adhesive.

From the wood failure, it can be seen that in the wet shear strength test at 63 °C for 3 h, the wood failure rate increases with the addition of glyoxylic acid. In the samples modified with 40% and 60% glyoxylic acid (S40 and S60), the wood failure rates are as high as approximately 70% and 90%, respectively. This phenomenon indicates that during the curing process, the adhesive forms a good bonding interface with the wood, effectively transferring stress, thus causing the wood to break rather than the adhesive layer during the shear process.

### 3.7. Water Resistance Analysis

**Figure 9:**
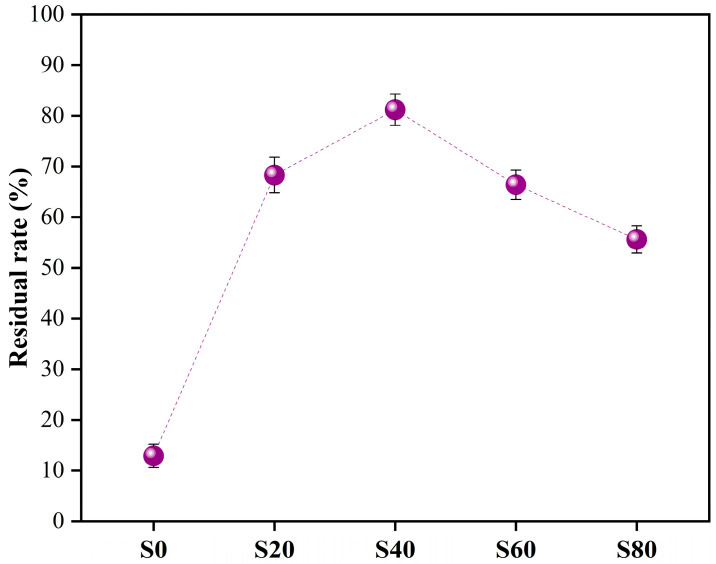
Residual rate of the curing product of sucrose-glyoxylic acid adhesive.

Sucrose-glyoxylic acid, as an environmentally friendly wood adhesive, has its water resistance as a key indicator to assess its potential for practical applications. The residual rate of the cured adhesive product is a more effective and direct method to measure its water resistance. By measuring the residual rate of the adhesive after hot water treatment, one can intuitively evaluate its stability in a water environment. As shown in Figure 9, the residual rate of the S0 sample is the lowest after hot water immersion, at only 12.9%. This indicates that the unmodified sucrose adhesive has poor water resistance and is prone to dissolution in warm water environments. The unmodified sucrose molecules are primarily bonded by hydrogen bonds, forming a weak cross-linked structure that cannot effectively resist the penetration and dissolution of water molecules.

With increase in the amount of glyoxylic acid added, the water resistance of the adhesive first improves and then declines. Specifically, when the amount of glyoxylic acid added is 20%, the residual rate of the adhesive increases to 68.3%; further increasing the amount of glyoxylic acid to 40% raises the residual rate to 81.2%; however, when the amount of glyoxylic acid continues to increase to 60% and 80%, the residual rates decrease to 66.4% and 55.6%, respectively. This phenomenon can be attributed to the cross-linked reactions between the aldehyde group and carboxyl group in glyoxylic acid and the hydroxyl group in sucrose molecules. These reactions form acetal structures and ester linkages, constructing a more stable three-dimensional network structure, thereby enhancing the cohesive strength of the adhesive and its bonding strength with the wood interface. This cross-linked structure not only improves the mechanical properties of the adhesive but also enhances its resistance to water molecules, making the chemical bonds in the cross-linked network more difficult to break in a water environment, thereby improving the water resistance of the adhesive. The adhesive modified with 40% glyoxylic acid has the highest residual rate, reaching 81.2%, indicating that an addition of 40% glyoxylic acid is the optimal choice for optimizing water resistance. At this addition level, the cross-linked reaction is the most complete, and the formed network structure is the most dense, thus providing the best water resistance. The SEM and XRD analysis results further reveal the effects of glyoxylic acid modification on the microstructure of the adhesive. The modified adhesive particles display a more regular block-like cross-linked structure, with distinct wrinkles and wavy patterns on the cross-sectional surface and a more compact texture. These changes in microstructure directly lead to significant improvements in macroscopic properties, including enhanced water resistance. When the amount of glyoxylic acid added exceeds 40%, the residual rate decreases. This may be due to the overly dense cross-linked network caused by the excess glyoxylic acid, which in turn affects the movement and reactivity of the molecular chains, thereby reducing the overall performance of the adhesive. Moreover, when the content of glyoxylic acid is high, the acidity of the system increases, and sucrose may produce sugar derivatives during the high-temperature curing process, adding some unreacted small molecules to the system. These small molecules can dissolve in hot water, thereby affecting the water resistance of the adhesive.

**Figure 10:**
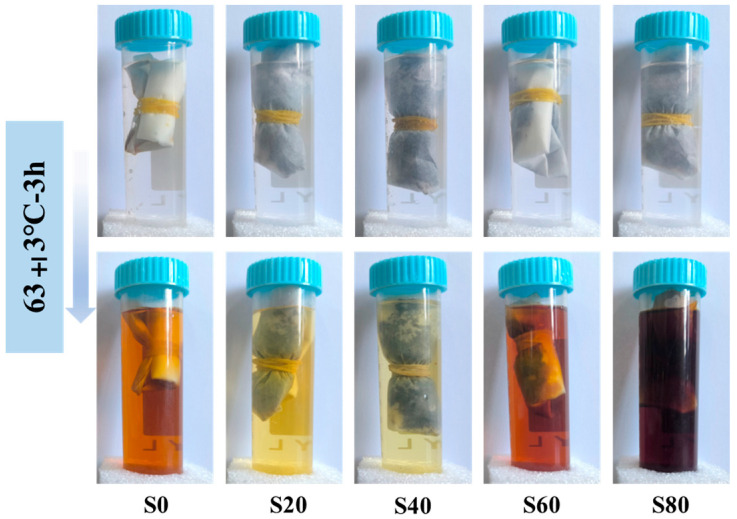
Photos of the cured product of the sucrose-glyoxylic acid adhesive before and after being immersed in warm water.

In addition, the appearance changes in the samples before and after immersion in warm water (Figure 10) can further confirm the changes in the water resistance of the adhesive. After being immersed in hot water at 63 ± 3 °C for 3 h, the S0 sample shows a noticeable change in the color of the solution to a light yellow. This phenomenon can be attributed to the partial degradation of sucrose during the high-temperature curing process, resulting in the formation of colored derivatives. These derivatives, along with the large number of hydrophilic groups in sucrose itself, can dissolve into the water solution, thus giving the water solution a light yellow color and resulting in a low residual rate.

In contrast, when the amount of glyoxylic acid added is 20% and 40%, the color of the water solution is significantly lighter due to the effective cross-linked reactions between the hydrophilic groups of sucrose and glyoxylic acid. This result further validates the positive role of glyoxylic acid modification in enhancing the water resistance of sucrose-based adhesives. However, when the amount of glyoxylic acid added increases to 60% and 80%, the color of the water solution gradually deepens. In terms of solution transparency, S0 is comparable to S60, while when the amount of glyoxylic acid added increases to 80%, the color and transparency of the water solution increase significantly. This phenomenon confirms the above speculation that excess acid can make sucrose more prone to forming sugar derivatives at high temperatures.

### 3.8. Hygroscopicity Analysis

**Figure 11:**
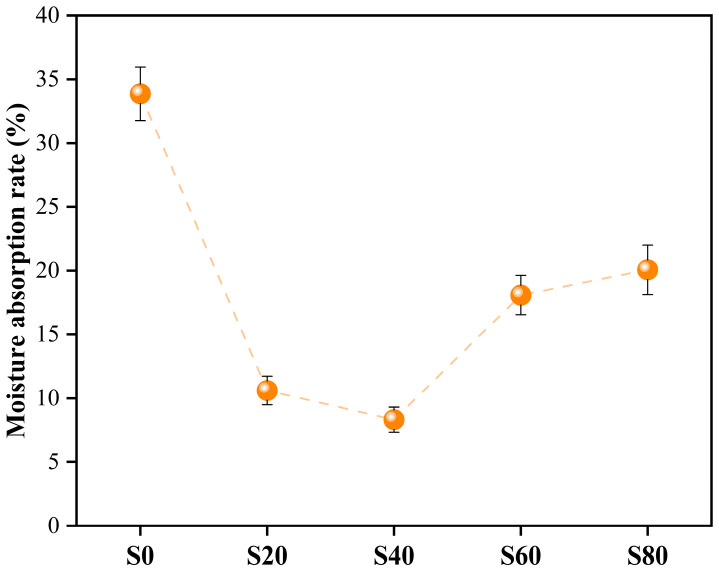
Hygroscopicity of the curing product of sucrose-acetohydroxy acid adhesive.

The hygroscopicity of adhesives is an important indicator for measuring their bonding stability in humid environments. Lower hygroscopicity means that the adhesive can maintain better performance under humid conditions and is less likely to experience a decrease in bonding strength due to moisture absorption. As shown in Figure 11, the unmodified sucrose adhesive has a high hygroscopicity, reaching 33.86%. This high hygroscopicity is mainly due to the large number of hydrophilic hydroxyl groups in sucrose molecules. These hydroxyl groups can form hydrogen bonds with water molecules, causing the adhesive to absorb a large amount of moisture in humid environments, thereby significantly reducing its bonding strength and overall performance.

However, as the amount of glyoxylic acid added increases, the hygroscopicity of the adhesive decreases significantly. Specifically, the hygroscopicity of the adhesive modified with 20% glyoxylic acid is 10.61%, while that of the adhesive modified with 40% glyoxylic acid further decreases to 8.32%, the lowest value among all samples. This result indicates that glyoxylic acid modification can effectively reduce the hygroscopicity of the adhesive, significantly enhancing its stability in humid environments, which is of great significance for the practical application of wood adhesives.

The effect of glyoxylic acid modification on the hygroscopicity of the adhesive is also attributed to the cross-linked reactions between glyoxylic acid and sucrose molecules, forming acetal structures and ester linkages, and constructing a more stable three-dimensional network structure. This cross-linked structure not only reduces the number of free hydroxyl groups in the adhesive, thereby decreasing its ability to form hydrogen bonds with water molecules and reducing moisture absorption, but also makes the microstructure of the modified adhesive more compact, with lower porosity, further preventing the penetration of moisture. These changes in microstructure directly lead to significant improvements in macroscopic properties, including reduced hygroscopicity. However, when the amount of glyoxylic acid added exceeds 40%, the hygroscopicity increases slightly. This may be due to the overly dense cross-linked network, which affects the movement and reactivity of the molecular chains, leading to incomplete cross-linked reactions or some side reactions. At the same time, the increased acidity of the system may cause sucrose to produce sugar derivatives during the high-temperature curing process. These derivatives may contain hydrophilic groups, increasing the hygroscopicity of the adhesive. Therefore, an addition of 40% glyoxylic acid is the optimal choice for optimizing the water resistance and hygroscopicity of the adhesive. At this addition level, the cross-linked reaction is the most complete, and the formed network structure is the most dense, thereby providing the best water resistance and the lowest hygroscopicity, ensuring the excellent performance of the adhesive in humid environments.

## 4. Conclusions

1. This study developed a high-performance bio-based wood adhesive through one-step esterification/acetalization of sucrose with glyoxylic acid. Optimal properties were achieved at 40% glyoxylic acid content, yielding wet shear strengths of 1.39 MPa (63 °C) and 1.17 MPa (93 °C) that substantially exceeded GB/T 17657-2022 requirements, while reducing hygroscopicity to 8.32% and elevating thermal decomposition temperature to 333.5 °C.
2. These improvements stemmed from formation of a dense, amorphous, three-dimensional cross-linked network that consumed hydrophilic hydroxyl groups and enhanced cohesive strength. FTIR confirmed ester and acetal linkages, while XRD/SEM revealed transformation from crystalline to compact amorphous morphology. The modified adhesive also reduced curing temperature to 202.8 °C, offering energy-efficient processing advantages.
3. Utilizing low-cost, abundant feedstocks via a streamlined synthesis, this adhesive demonstrates strong industrial viability as a sustainable alternative to petroleum-based resins. However, the narrow optimal formulation window (>40% glyoxylic acid caused degradation) and lack of scalability studies represent key limitations. Future work should focus on process optimization, long-term durability evaluation, and economic feasibility assessment to facilitate commercial adoption. This research advances both our fundamental understanding of sucrose-based crosslinking chemistry and the practical development of eco-friendly adhesives for wood industry decarbonization.

## Figures and Tables

**Table 1:** Adhesive formula information.

Samples	Sucrose (g)	Deionized Water (g)	Glyoxylic Acid (g)	pH Before Curing
S0	30	30	0	7.0
S20	30	30	12	5.0
S40	30	30	24	3.0
S60	30	30	36	2.0
S80	30	30	48	1.5

## Data Availability

The original contributions presented in this study are included in the article. Further inquiries can be directed to the corresponding authors.
